# Cross-Talk Between Tumor Cells Undergoing Epithelial to Mesenchymal Transition and Natural Killer Cells in Tumor Microenvironment in Colorectal Cancer

**DOI:** 10.3389/fcell.2021.750022

**Published:** 2021-11-10

**Authors:** Ana Vuletić, Katarina Mirjačić Martinović, Nevena Tišma Miletić, Jerome Zoidakis, Sergi Castellvi-Bel, Milena Čavić

**Affiliations:** ^1^Department of Experimental Oncology, Institute of Oncology and Radiology of Serbia, Belgrade, Serbia; ^2^Department of Biotechnology, Biomedical Research Foundation, Academy of Athens, Athens, Greece; ^3^Gastroenterology Department, Hospital Clínic, Centro de Investigación Biomédica en Red de Enfermedades Hepáticas y Digestivas (CIBERehd), Institut d’Investigacions Biomčdiques August Pi i Sunyer (IDIBAPS), University of Barcelona, Barcelona, Spain

**Keywords:** NK cells, tumor microenvironment, NK cell receptors, epithelial to mesenchymal transition, colorectal cancer

## Abstract

Tumor cells undergoing epithelial to mesenchymal transition (EMT) and immune cells in tumor microenvironment (TME) reciprocally influence each other. Immune cells, by supplying TME with bioactive molecules including cytokines, chemokines, enzymes, metabolites, and by physical interactions with tumor cells via their receptors, represent an important factor that affects EMT. Chronical inflammation in TME favorizes tumor growth and invasiveness and stimulates synthesis of EMT promoting transcription factors. Natural killer (NK) cells, owing to their unique ability to exert cytotoxic function independent of major histocompatibility (MHC)-mediated antigen presentation, play a significant role in the control of metastasis in colorectal cancer (CRC). Although, the cross-talk between immune cells and tumor cells in general favors the induction of EMT and inhibition of antitumor immune responses, there are some changes in the immunogenicity of tumor cells during EMT of CRC cells that increase their susceptibility to NK cell cytotoxic lysis. However, suppressive TME downmodulates the expression of activating NK cell receptors, decreases the expression of activating and increases the expression of inhibitory NK cell ligands on tumor cells, and impairs NK cell metabolism that altogether negatively affects the overall NK cell function. Furthermore, process of EMT is often associated with increased expression of programmed cell death ligand (PD-L) and expression of immune checkpoint molecules PD-1, TIGIT, and TIM3 on functionally exhausted NK cells in TME in CRC. In this review we discuss modalities of cross-talk between tumor cells and NK cells, with regard of EMT-driven changes.

## Introduction

Natural killer (NK) cells are a distinct population of innate lymphoid cells that are able to eliminate malignantly transformed, damaged or infected cells by cytotoxic lysis. Unlike cytotoxic T cells, NK cells have a unique ability to directly recognize stressed cells via their germline encoded receptors and promptly exert cytotoxic function without major histocompatibility (MHC) class I molecule restriction (Caligiuri, 2008). NK cells are divided into two functional subsets, according to the expression density of their surface marker, CD56 neural adhesion molecule: low density of CD56 i.e., cytotoxic CD56^dim^ with abundant perforin and granzyme granules, and high density of CD56 the predominantly regulatory subset CD56^bright^ with the ability to produce interferon-gamma (IFN-γ), tumor necrosis factor (TNF), interleukin (IL)-10, IL-13, and granulocyte-macrophage colony-stimulating factor (GM-CSF). In this sense, NK cells also promote adaptive immune responses by secreting cytokines (Cooper et al., 2001; Vivier et al., 2011).

NK cell-mediated antitumor response is tightly regulated by the balance of signals transmitted by activating and inhibitory receptors and their ligands on tumor cells. NK cells have the ability to distinguish stressed cells (infected, tumor, etc.) from healthy cells by the two complementary recognition strategies that can be termed as “missing self” and “stress induced self-ligands” (Ljunggren and Kärre, 1990; Jurišić et al., 2020).

NK cells express inhibitory killer cell immunoglobulin-like receptors (KIR)s that inhibit NK cell cytotoxic activity toward “normal” i.e., healthy cells that express MHC class I molecules (Campbell and Purdy, 2011). According to “missing self” hypothesis, activation of NK cells occurs in contact with malignant cells that have lost MHC class I molecules and have become susceptible to NK cell lysis (Ljunggren and Kärre, 1990). Other important groups of inhibitory receptor that have binding affinity toward MHC molecules are c-type lectin receptor that consists of CD94-NKG2A heterodimer and specifically binds to non-classical MHC class Ib molecules (i.e., HLA-E) (Iwaszko and Bogunia-Kubik, 2011), leukocyte Ig-like receptors (LIR) that recognize classical MHC class I molecules, and also interact with non-classical MHC class I and bacterial proteins with low binding affinities (Heidenreich et al., 2012). Inhibitory KIR (KIRDL) receptors suppress NK cell activity through a receptor-associated immune tyrosine based inhibitory motif (ITIM) by recruiting protein tyrosine phosphatases (SHP-1 and SHP-2) responsible for dephosphorylation of tyrosine kinases associated with activating NK cell receptors (Campbell and Purdy, 2011). There are also some inhibitory NK cell receptors that are able to recognize non-MHC ligands like killer cell lectin-like receptor G1 (KLRG1) that interacts with cadherin adhesion molecules (Farag and Caligiuri, 2006; Konjević et al., 2016; Müller-Durovic et al., 2016).

NK cell cytotoxicity can be activated in contact with malignant cells that express stress-induced ligands (“stress induced self”) that bind to activating NK cell receptors. Activating NK cell receptors include several receptor families such as natural cytotoxicity receptor (NCR) family, NKp46, NKp30, and NKp44, the C-type lectin receptor family [natural killer group 2D (NKG2D), CD94/NKG2C, CD94/NKG2E, CD94/NKG2F], costimulatory receptor CD226 (DNAX accessory molecule-1, DNAM1), natural killer receptor-P1 (NKR-P1 family) CD161, activating receptors that belong to the KIR family (KIR2DS1, KIR2DS4, and KIR2DL4), and Fc fragment binding receptor IIIA (CD16) that cooperate and determine NK cell cytotoxicity against transformed cells (Farag and Caligiuri, 2006; Hudspeth et al., 2013; Konjević et al., 2016). Most of the activating receptors transmit signals through the phosphorylation of the tyrosine (Tyr) residue in specific Tyr-based structural motifs (ITAM)s (Purdy and Campbell, 2009). NKG2D is a particularly relevant activating receptor that recognizes a group of MHC class I polypeptide-related sequence A and B (MICA and MICB) and UL16 binding protein molecules (ULBP1-6), which are stress-inducible molecules expressed on malignantly transformed cells (López-Larrea et al., 2008; Zafirova et al., 2011).

Growing knowledge indicates that aside from inhibitory and activating KIRs, some receptor families are composed of paired receptors that, in spite of binding to similar ligands, have opposite, activating, or inhibitory function. In this sense a typical example is a family of nectin-binding adhesion molecules that includes activating receptor CD226 (DNAX accessory molecule-1, DNAM1), and its inhibitory counterparts CD96 [T cell-activated increased late expression (TACTILE)] and T-cell immunoglobulin and ITIM domain (TIGIT) receptors. These receptors bind nectin proteins, CD112 (nectin-2), and CD155 [poliovirus receptor (PVR)] and have been recently identified as crucial regulators of NK cell function (Martinet and Smyth, 2015; Konjević et al., 2017a).

NK cells are not only found in peripheral blood (PB) but also populate different organs and tissues as blood-tissue circulating or tissue-resident, and participate in immunosurveillance of disseminated cancer cells (Vitale et al., 2020). NK cells are capable of killing multiple tumor targets that enter into circulation and are also able to enter the tumor site by extravasation through the tumor vasculature. The major chemokine receptor involved in NK cell migration toward the tumor is CXCR3 that binds to the tumor-derived chemokine (C-X-C motif) ligands CXCL9, 10, and 11 (Wennerberg et al., 2014, 2015). In colorectal cancer (CRC), increased CXCL10 expression has been found in tumor tissue compared to the adjacent normal tissue (Zou et al., 2020). It has been shown that in the settings of therapy of CRC with adoptive cell transfer, mostly CXCR3-positive expanded NK cells infiltrate tumors (Wennerberg et al., 2015). However, in CRC NK cells are mostly present in adjacent mucosa and organ stroma but not in direct contact with tumor cells despite of the high level of local chemokines (Halama et al., 2011). Tumor infiltrating NK cells in most tumors, including CRC, are usually low cytotoxic CD56^bright^ or poorly functional CD56^dim^ (Levi et al., 2015), and according to one study able to secrete proangiogenic cytokines and proangiogenic factors (Bruno et al., 2018). Therefore, the scarce number of NK cells in the tissue of solid tumor and their poor functionality represent the considerable obstacle for unambiguous conclusion weather tumor infiltrating NK cell are relevant for disease prognosis. Some studies have reported the longer disease-free survival of CRC patients with increased NK cell infiltration of the tumor (Coca et al., 1997; Menon et al., 2004), while several studies showed no effect of NK cell inflation for disease prognosis (Sandel et al., 2005; Halama et al., 2011). Studies investigating the joint effect of immune cell subsets in the tumor milieu on disease prognosis in CRC have shown that the presence of both NK cells and CD8^+^ T cells in CRC has a favorable prognostic impact (Sconocchia et al., 2014; Coppola et al., 2015).

The majority of tumors lack or express low levels of MHC class I molecule and therefore escape T cell immune surveillance. NK cells are present in peripheral blood, lymph nodes, and may owing to their unique ability to display MHC I-independent cytotoxicity play an important role in immunosurveillance of disseminated tumor cells in tumor draining lymph nodes (Vuletić et al., 2013; Ali et al., 2014) and in eradication of distant metastasis (Lakshmikanth et al., 2009; Lorenzo-Herrero et al., 2018). However, in malignancies tumor-derived immunosuppressive factors often affect the expression of NK cell receptors that together with cytolytic molecule dysregulation, leads to inhibition of NK cell function (Konjević et al., 2012, 2017a).

### Regulation of Epithelial to Mesenchymal Transition

The metastatic cascade involves detachment of tumor cells from surrounding cells, local invasion of surrounding tissue and tumor cell entrance into nearby vasculature. Concurrently, tumor cells partially lose epithelial markers; acquire mesenchymal-like phenotype and migratory i.e., invasive properties during the process of epithelial to mesenchymal transition (EMT). Detached tumor cells travel via bloodstream or lymphaticum, extravasate via transendothelial migration and invade distant tissues and organs. The ability of certain tumors to form metastasis is a result of a plethora of factors. Intrinsic i.e., tumor cell-associated factors such as epigenetic changes and signaling pathways activated by oncogenic mutations are submitted to selective pressure and shaped by tumor-extrinsic factors such as hypoxia, low pH, antitumor drugs, host’s antitumor immune responses, growth factors, cytokines and other bioactive molecules in tumor microenvironment (TME) that favorize specific malignant traits (Fedele and Melisi, 2020). Cytokines and immunosuppressive mediators in TME are produced by tumor cells, cells of organ stroma, including vasculature, fibroblasts, and by immune cells which create an inflammatory environment that further affects all resident and infiltrating cells. In this sense, TME induces differentiation of fibroblasts into cancer associated fibroblasts (CAF)s which also produce cytokines, growth factors and further potentiate the immunosuppressive and growth promoting immune milieu of tumors (Schiavoni et al., 2013). Inflammation in TME contributes to tumor growth and progression. Accordingly, EMT is also influenced by a crosstalk between tumor cells and the cells in the TME, including immune cell subsets (Romeo et al., 2019; Fedele and Melisi, 2020).

In the TME, a multitude of different cell types are present: cells of innate immune system [dendritic cells (DC)s, macrophages, NK cells], cells of adaptive immune system (T and B cells) and suppressive cells of immune system [myeloid-derived suppressor cells (MDSC)s, regulatory T cells (Treg)s, and immunosuppressive subset of macrophages M2, tumor associated macrophages (TAMs)], CAFs as well as tumor cells and residing cells of organ tissue. All these cells produce cytokines, growth factors and other mediators that induce EMT of tumor cells and also affect function of tumor infiltrating NK cells. A number of inflammatory cytokines in TME induce EMT: interleukin (IL)-6 produced by a variety of different cell types such as fibroblasts, endothelial cells, macrophages and T cells, IL-8 produced by macrophages, endothelial cells fibroblasts, and tumor cells, TNF produced by the cells of the innate immune systems (macrophages, NK cells), activated T cells, fibroblasts and endothelial cells, transforming growth factor beta (TGF-β) produced by all immune cells but most abundantly by MDSCs, Tregs, and tumor cells, and IL-10 produced by macrophages, DCs, B cells, NK cells, Tregs, and tumor cells (De Simone et al., 2015; Konjević et al., 2017a, 2019; Fedele and Melisi, 2020). The process of EMT is accompanied with decreased infiltration of immunoreactive immune cells i.e., tumor infiltrating lymphocytes (TIL)s, and increased infiltration of suppressive immune cells into the TME (López-Soto et al., 2017).

### Changes in Tumor Immunogenicity and Susceptibility to Natural Killer Cell Antitumor Activity During Epithelial to Mesenchymal Transition

**FIGURE 1:**
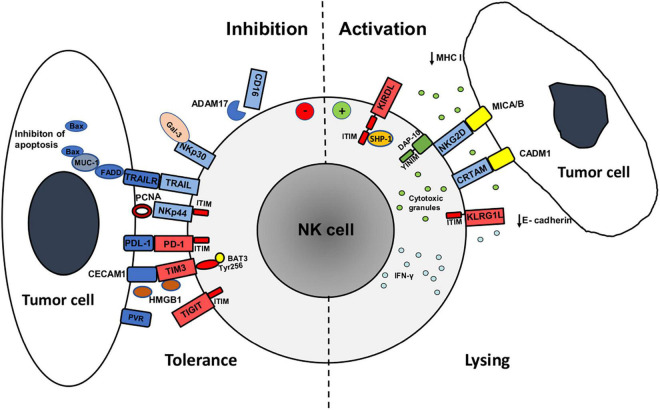
Mechanisms of activation and inhibition of NK cell antitumor activity against tumor cell undergoing epithelial to mesenchymal transition (EMT). The expression of major activating (blue) and inhibitory (red) receptors by NK cells and their ligands on tumor cells determines antitumor activity (cytotoxicity and IFN-γ production). Tumor cells during EMT downregulate the expression of MHC class I and E-cadherin ligands for inhibitory receptors KIRDL (Killer cell immunoglobulin like receptors long cytoplasmic tail) and KLRG1 (Killer Cell Lectin Like Receptor G1), respectively, and upregulate MICA/B and CADM1 ligands for activating receptors NKG2D and CRTAM, respectively, that increases tumor cell susceptibility to NK cell activity. Increased expression of PCNA and galectin-3 ligands for NKp44 and NKp30 receptors, respectively, upregulation of immune checkpoint receptors (PD-1, TIM3, TIGIT) on NK cells and upregulation of PD-1 ligand (PDL-1) and TIM3 ligands (CECAM1, HMGB1) during EMT, have negative effect on NK cell function. Moreover, the expression of MUC-1 protein in tumor cells inhibits NK cell killing of EMT tumor cells via TNF related apoptosis inducing ligand (TRAIL) via inhibition of Bax dimerization. NK cells upregulate the expression of PD-1, TIM3, and TIGIT immune checkpoint receptors that alongside with matrix metalloproteinase ADAM17- induced CD16 receptor shedding in tumor microevent during EMT reduces NK cell activity. Structural motifs involved in activating signaling are green while inhibitory are red: Tyrosine-based motif (YINM) structural motifs and DNAX-activating protein (DAP10) are associated with NKG2D and induce NK cell activation. Immunoreceptor tyrosine-based inhibitory (ITIM) structural motifs are associated with KIRDL, KLRG1, TIGIT, and NKp44 (when ligated to PCNA) receptors during NK cell inhibition. Inhibitory receptor KIRDL suppresses NK cell activity ITIM by recruiting protein tyrosine phosphatase (SHP-1). TIM-3 receptor inhibitory signaling is mediated through Tyrosine 256 (Ty256) structural motif that interacts with HLA-B-associated transcript 3 (BAT3). Fas-associated death domain (FADD) is transmits TRAIL and TRAIL receptor (TRAILR) signaling pathway that is during EMT inhibited by MUC-1 binding that prevents Bax dimerization and apoptosis of tumor cell.

EMT is associated with changes in immunogenicity of tumor cells in terms of regulation of surface molecules that are either directly or indirectly involved in immune recognition of tumor cells. During acquisition of mesenchymal-like properties tumor cells often show reduced expression of tumor antigens and immunoproteasome components that altogether lead to reduced presentation of antigenic peptides. Most importantly, tumor cells undergoing EMT decrease the expression of MHC class I molecules, making them resistant to CD8^+^ T cell cytotoxicity, but more susceptible to NK cell lysis (Tallerico et al., 2013; Ferretti et al., 2020; Melaiu et al., 2020; Figure 1). These changes are associated with the prolonged, i.e., chronic, inflammation in TME, as opposed to the protective, acute inflammation during the early phase of antitumor immune response when type I interferons produced by antigen presenting cells (DCs) and IFN-γ produced by T and NK cells in TME increase the expression of MHC molecules on tumor cells. Prolonged inflammation leads to loss of MHC I expression on tumor cells and can be attributed to the effect of an immunosuppressive cytokine TGF-β, IL-10, and the epidermal growth factor (EGF) which are secreted in the TME (Stanilov et al., 2010; Chen et al., 2015; Lorenzo-Herrero et al., 2018; Fedele and Melisi, 2020).

Cytokines and growth factors in TME stimulate synthesis of transcription factors that further induce mesenchymal phenotype and subsequent changes of immunogenicity of tumor cells during EMT. There are several major groups of transcription factors involved in EMT: SNAIL family of zinc-finger transcription factors Snail/Slug, the zinc finger E-box binding homeobox (ZEB) family of transcription factors ZEB1/ZEB2, and the TWIST family of basic helix-loop-helix (bHLH) transcription factors Twist1/Twist2, and DNA-binding forkhead box (FOX) transcription factors (Vu and Datta, 2017). In CRC the aberrant regulation of EMT-related transcription factors is associated with increased rate of cancer recurrence and decreased survival of CRC patients. Previous studies have reported that overexpression of EMT-related transcription factors, such as Snail, Slug, Twist1,2, ZEB1/ZEB2, and FOXC2, FOXQ1, FOXC1, and FOXM1 is associated with invasiveness, metastasis, and poor prognosis of CRC (Francí et al., 2009; Gomez et al., 2011; Zhang et al., 2013; Lu et al., 2018).

Loss of E-cadherin expression, one of the principal organizers of the epithelial phenotype and an important component for cell adherence, is a major hallmark of EMT that enables tumor cells to migrate and form metastatic sites (López-Soto et al., 2013; Chockley et al., 2018; Figure 1). It has been shown that TGF-β, via its signal transducer Smad protein that is associated with TGF-β receptor, induces expression of high mobility group A2 (HMGA2) protein which regulates expression of many important repressors of E-cadherin transcription (Thuault et al., 2006). Smads and HMGA2 cooperatively bind to the Snail promoter and induce Snail expression, E-cadherin repression, and the overall EMT phenotype (Thuault et al., 2008). In this sense, TGF-β and EGF signaling in the TME induces loss of epithelial marker E-cadherin, which is a ligand for the KLRG1 inhibitory NK cell receptor, and increases expression of mesenchymal N-cadherin. This contributes to increased susceptibility of tumor cells undergoing EMT to NK cell-mediated lysis. Aside from Snail 1, ZEB1, and ZEB2 are also involved in suppression of E-cadherin transcription (Qin et al., 2016). Furthermore, it has been shown first in pancreatic cancer and latter in CRC, that proangiogenic factor VEGF induces and E- to N- cadherin switch by increasing the expression of Snail, Twist, and Slug EMT-associated transcription factors (Yang et al., 2006; Bendardaf et al., 2019).

During progression of EMT in CRC cells, TGF-β stimulation via induction of Snail1 transcription factor up-regulates the expression of ligands for activating NKG2D receptor (NKG2DL) s in epithelial cells, rendering EMT cells more susceptible to NK cell-mediated killing (López-Soto et al., 2013). In this sense, activation of Sp1 and Sp3 transcription factors which are key regulators of transcription of MICA/B and ULBP1–3 NKG2DLs has been observed during the process of EMT. In cells of healthy colon mucosa and in well-differentiated CRC, MICA/B and ULBP1 NKG2DLs are expressed mostly on the luminal part of the epithelial layer, whereas a loss of the polarization of NKG2DL expression was observed in malignant cells during the advancement of EMT (López-Soto et al., 2006; Huergo-Zapico et al., 2014). This fact has been found to coincide with an increase in the number of NKG2D positive tumor-infiltrating lymphocytes, resulting in their elimination by NKG2D-bearing immune cells. These findings suggest that the loss of the epithelial integrity and polarity may result in diffusion of MICA/B proteins along the membrane of tumor cells during acquisition of mesenchymal-like phenotype. Overall, these data suggest that tumor progression and metastasis in CRC, may also be under the control of the NKG2D response (Huergo-Zapico et al., 2014). Furthermore, it has been shown in CRC cell lines that signal transducer and activator of transcription 3 (STAT3) is a negative regulator of MICA transcription and that the upregulation of MICA ligand on tumor cells correlates with dephosphorylation of STAT3 (Bedel et al., 2011; López-Soto et al., 2013).

There are multiple phenotypic changes of tumor cell during EMT that involve alterations in the expression of ligands for diverse activating NK cell receptors, including upregulation of PVR ligand for DNAM1 receptor that was shown in breast and lung cancer (Chockley et al., 2018). Furthermore, process of EMT is associated with upregulated expression of cell adhesion molecule 1 (CADM1), a recently identified NK cell ligand, that binds to the cytotoxic and regulatory T cell–associated molecule (CRTAM) receptor (Boles et al., 2005; Figure 1). In this sense, upregulation of CADM1 along with downregulation of E-cadherin on tumor cells undergoing EMT are major factors that contribute to NK cell-mediated immunosurveillance of metastasis (Chockley et al., 2018).

However, the expression of some surface and extracellular molecules that bind to activating NK cell receptors during EMT reduces tumor cells susceptibility to NK cell lysis (Figure 1). This is the case with increased expression of galectin-3, a beta-galactoside-binding protein, which inhibits adhesion between tumor cells and with extracellular matrix, and therefore promotes cell mobility and tumor invasiveness (Liu and Rabinovich, 2005; Farhad et al., 2018). Binding of galectin-3 to activating NK cell receptor NKp30 suppresses NK cell-mediated tumor lysis (Wang et al., 2014; Krijgsman et al., 2020). High galectin 3 expression was found in CRC undergoing EMT and correlated with the poor clinical outcome (Endo et al., 2005). Moreover, as NKp30 has three different isoforms, NKp30 a, b, and c, it has been shown that binding of NKp30c to its ligand induces an immunosuppressive signal by producing IL-10 that subsequently reduces NK cell effector functions. Therefore, the final outcome of NKp30 activation depends on the presence of ligands on target cells, as well as of the presence of activating (a and b) or inhibitory (c) receptor isoforms on the surface of NK cells (Hudspeth et al., 2013). Similarly to galectin-3, the expression of proliferating cell nuclear antigen (PCNA) that is associated with enhanced tumor cell proliferation and invasive potential, leads to the inhibition of NK cell function via binding to activating NCR receptor NKp44 (Rosental et al., 2011; Rusakiewicz et al., 2017; Krijgsman et al., 2020). Binding of PCNA to NKp44 receptor inhibits NK cells cytotoxic activity and IFN-γ secretion and this inhibition is mediated by an ITIM structural motif of the NKp44 cytoplasmic domain (Rosental et al., 2011, 2012).

Aside from classical MHC class I molecules, the expression of ligands for inhibitory KIRs may be altered during EMT. In this sense, HLA-G, a non-classical MHC class I antigen that is expressed under physiological conditions in a few immune-privileged tissues including cytotrophoblast, can be induced in tumors. NK cells recognize HLA-G via KIR receptor 2DL4 (KIR2DL4 or CD158d) and LIR receptors B1 (LILRB1, ILT2, or CD85j) and B2 (LILRB2, ILT4, or CD85d). The aberrant induction of HLA-G expression is observed in most cancer histological types and it has been related to tumor metastasis and poor clinical prognosis. HLA-G expression has been shown in CRC although its relation to EMT has not been resolved, yet (Lin and Yan, 2018*).* The inhibitory receptor CD94/NKG2A and its ligand HLA-E are frequently overexpressed in many types of tumors, including CRC (Zhen et al., 2013; Cózar et al., 2021).

Tumor cells that express an EMT-promoting brachyury transcription factor often show upregulated expression of transmembrane protein MUC-1 which decreases tumor cell susceptibility to TNF related apoptosis inducing ligand (TRAIL) apoptosis and tumor susceptibility to perforin/granzyme-dependent lysis by CTLs and NK cells (David et al., 2016; Romeo et al., 2019). Circumstantial evidence for this phenomenon has been shown in the context of MUC1 inhibition by siRNA- gene silencing (David et al., 2016). Possible mechanism of the inhibition of TRAIL pathway by MUC-1 reported in one study involves the inhibition of cleavage and activation of caspase-8 (Agata et al., 2008), while another showed that MUC1 inhibits apoptosis by preventing dimerization of the pore-forming protein BAX and hence by preserving mitochondrial integrity (Ahmad et al., 2012). In the agreement with the latter, studies of MUC1 gene knockdown showed the loss of mitochondrial transmembrane potential in response to TRAIL (David et al., 2016). In CRC, MUC-1 expression has been shown to correlate with nodal metastasis and is used as a predictive factor for the emergence of distant metastasis (Zeng et al., 2015).

### Immune Checkpoints in Natural Killer Cell-Epithelial to Mesenchymal Transition Cross-Talk

As a consequence of immune responses, immune cells upregulate the expression of inhibitory checkpoint molecules whose primarily physiological role is to prevent excessive immune responses. There is a number of immune checkpoint molecules that contribute to immunosuppression in cancer and are upregulated on T and NK in TME as a consequence of chronic inflammation: programmed cell death receptor (PD)-1, cytotoxic T lymphocyte antigen (CTLA)-4, T cell immunoglobulin and mucin domain-containing protein 3 (TIM3), lymphocyte activation gene-3 (LAG-3), and TIGIT (Beldi-Ferchiou and Caillat-Zucman, 2017). The infiltration of suppressive immune cells into TME and ongoing secretion of immunosuppressive cytokines that favor EMT of tumor cells, contribute to upregulation of inhibitory immune checkpoint molecules on T and NK cells. Invasive phenotype of tumor cells and process of EMT are associated with increased expression of ligands for immune checkpoint receptors on tumor cells (Romeo et al., 2019; Figure 1).

During tumorigenesis oncogenic pathways, genetic, and epigenetic factors intrinsic to tumor cells upregulate the expression of PD-1 ligands (L)1 (B7H1), and L2 (PD-L2) on tumor cells. In this sense, mitogen-activated protein kinases (MAPK), phosphoinositide 3-kinase (PI3K), Janus kinase (JAK)/STAT3, and phospholipase Cγ signaling have been related to the regulation of PD-L1 expression in CRC (Li et al., 2019). Extrinsic factors in TME, including cytokines IFN-γ, IL-6, TNF, further favorize cancer immune escape by augmenting PD-L1 expression (Gao et al., 2018; Ju et al., 2020). The gene promoter region of PD-L1 gene also contains a binding site for ZEB1, an EMT-inducing transcription factor (Tsutsumi et al., 2017). Aside from tumor cells, multiple cells in TME, including immune cells (DCs, macrophages, Tregs) and CAFs also express PD-L1 (Curiel et al., 2003; Nazareth et al., 2007; Zou et al., 2016) that further reduces antitumor immunity. In this sense, PD-L1 is upregulated by IFN-γ on antigen-presenting cells (APCs) in TME and lymph nodes that leads to inhibition of T cell activation (Wang et al., 2018). Upregulation of PD-L1 expression has been found in metastatic CRC compared to primary tumors (Wang H. B. et al., 2017) and has been evaluated as prognostic factor in CRC (Chen et al., 2021). Although PD-L2 is predominantly expressed in immune cells, recent studies have detected its expression in CRC cells and determined that it was associated with poor patient survival (Wang H. et al., 2017; Chen et al., 2021). Therapeutic blockade of immune checkpoints with anti-PD-1 anti-PDL-1 antibodies has shown in recent years a considerable clinical benefit in oncology and has been introduced in treatment of metastatic CRC. As *c*onventional chemotherapeutics induce the expression of PD-L1 the novel combined therapeutic approaches in CRC have been introduced by focusing on targeting of PD-1/PD-L1 axis (Zou et al., 2016; Wang H. B. et al., 2017).

PD-1 is expressed on fully functionally mature CD56^dim^ NK cells and its expression is induced upon persistent stimulation of activating receptors (Pesce et al., 2017), while PD-1 mRNA splicing isoforms and cytoplasmic proteins are detectable in virtually all NK cells (Mariotti et al., 2018). High levels of PD-L1 expression were detected on tumors with low MHC class I expression (Šmahel, 2017; Ntomi et al., 2021) and implicate NK cells as non- MHC- restricted innate effectors in response to anti PD-1 therapy. In this sense, immune checkpoint blockade therapy with anti-PD-1 anti PDL-1 antibodies may also help to harness NK cell antitumor activity.

TIM3, a coinhibitory or immune checkpoint receptor, interacts with multiple ligands expressed on tumor cells such as HLA-B-associated transcript 3 (BAT3), carcinoembryonic antigen-related cell adhesion molecule (CEACAM)1, phosphatydilserine on apoptotic cells, galectin-9 that is present as surface molecule or in soluble form when secreted by tumor cells, and high mobility group protein B1 (HMGB1). The role of TIM3 expression on NK cell function remains controversial as binding of TIM3 to its ligand galectin-9 enhances IFN-γ production but has no effect on NK cell cytotoxicity (Wolf et al., 2020). However, TIM3^+^PD-1^+^ NK cells from late-stage cancer patients are less cytotoxic than TIM3^–^PD-1^–^ NK cells and TIM3 blockade subsequently restored NK cell functions (Seo et al., 2017). In tumors with high mesenchymal scores, increased levels of soluble lectin galectin-9 and the adhesion molecule CEACAM1, both ligands of TIM3, have been reported. TIM3 expression defines a subset of functionally exhausted NK cells (Seo et al., 2017; Cózar et al., 2021). In the context of HMGB1 ligand, that is released during application of chemotherapeutic drugs, it has been suggested that TIM3 blockade in combination with chemotherapy may alleviate immunosuppression and show some therapeutic benefit (Wolf et al., 2020).

TIGIT inhibitory checkpoint molecule competes with activating DNAM1 receptor for the same ligands on tumor cells and in CRC patients it is more highly expressed on intratumoral NK cells than on peritumoral NK cells (Zhang et al., 2018). Blockade of TIGIT with monoclonal antibody increases T cell activity in NK cell-dependent manner and shows synergistic effect with anti-PD1 therapy (Cózar et al., 2021). High TIGIT expression was correlated with NK-cell exhaustion in tumor bearing mice and patients with CRC (Zhang et al., 2018; Cózar et al., 2021).

Tumors with high mesenchymal scores show increased expression of B7H3 and CD47 (Mak et al., 2016). B7H3 immune checkpoint molecule, a member of B7/CD28 superfamily, exhibits inhibitory effects in modulating T cells and NK cell activity (Suh et al., 2003), although several studies have found a co-stimulatory role of B7-H3 in T cell activation and IFN-γ production (Ni and Dong, 2017; Yang et al., 2020). However, B7-H3 overexpression is associated with proliferation and invasive potential and EMT of CRC (Ingebrigtsen et al., 2012; Jiang et al., 2016) and was negatively associated with overall survival rate in CRC (Mao et al., 2017). CD47 surface antigen can be designated as “don’t eat me” as its expression on tumor cells upon binding to Signal regulatory protein α (SIRPα) in macrophages suppresses phagocytosis. Thus, tumor cells expressing CD47 can escape the antitumor effect of the innate immune system. CD47 is upregulated by EMT inducing transcription factors Snail1 or ZEB1 (Noman et al., 2018).

During the last decade immune checkpoint inhibitors (ICI) have proven to be promising agents in therapy of CRC. Pembrolizumab and nivolumab, ICIs that target PD-1, showed considerable antitumor activity in a subset of CRC patients with mismatch-repair-deficiency (dMMR) and high microsatellite instability (MSI-H) (dMMR/MSI-H), and have been officially approved for the treatment of these patients (Overman et al., 2017; Le et al., 2020). However, MSI-H/dMMR tumors account for only 5% of metastatic CRC, while in the remaining CRC patients identified as microsatellite stable/DNA mismatch repair proficient (MSS/pMMR), these agents have not shown any therapeutic benefit (Almquist et al., 2020). An important reason for the failure of ICI therapy is the acquisition of resistance, so that combinations of different ICI inhibitors have been investigated. In this sense, clinical trials evaluating the role of nivolumab in combination with CTLA-4 ICI (ipilimumab) conducted in metastatic MSI-H/dMMR CRC patients have shown therapeutic benefit (Overman et al., 2018). Currently, various preclinical and clinical trials evaluating ICIs in combination with growth factor (VEGF, EGFR) inhibitors, tyrosine kinase, e.g., MEK inhibitors, and IDO inhibitors and agonists (TLR, OX40, 4-1BB agonists) have been conducted (Almquist et al., 2020). At this time, further advances in the understanding of fine mechanisms of interactions between immunosuppressive TME and different molecular profiles of tumor cells, as well as biomarkers of ICI resistance, especially in MSS/pMMR tumors, would aid in determining the most effective therapeutic combination for the treatment of CRC.

### Tumor Microenvironment—Induced Effects on Natural Killer Cells

NK cells in TME are often characterized by the impairment of antitumor cytotoxic function and low ability to produce IFN-γ. Low NK cell cytotoxicity may originate from the low level of cytotoxic molecules (granzymes and perforin), and decreased expression of activating or increased expression of inhibitory NK cell receptors that shifts the balance of receptor-ligand signals to poor NK cell activation (Konjević et al., 2012).

Inflammatory cytokines in TME affect NK cell receptor repertoire and lead to progressive local and systemic inhibition of NK cell function. In this sense, the EMT-inducing cytokines TGFβ, IL-10, IL-6, produced by tumor cells or by immunosuppressive cells in TME, can either directly downmodulate expression of activating NK cell receptors (NKG2D, NCRs, DNAM1, CD16) or indirectly by inducing the differentiation of suppressive immune cells such as M2 macrophages, tolerogenic DCs, Tregs and MDSCs and their ability to produce additional immunosuppressive factors (Stojanovic et al., 2013; Konjević et al., 2016, 2017a, 2019). Suppressive cytokines, metabolites L-kynurenine, prostaglandine E2, nitric oxide (NO) produced by immunosuppressive enzymes indolamine-2,3-dioxygenase (IDO), cyclooxygenase 2 (COX2), inducible NO synthase (iNOS), respectively, and vascular endothelial growth factor (VEGF), produced by suppressive and regulatory immune cells and tumor cells, further downregulate the expression of activating NK cell receptors (Della Chiesa et al., 2006; Harizi, 2013; Konjević et al., 2017a; Park et al., 2018; Table 1). These factors generate a chronic inflammatory immunosuppressive milieu that contributes to the suppression of the antitumor function of NK cells (Schiavoni et al., 2013; Konjević et al., 2017a). Consequently, decreased expression of NKG2D, NKp30, NKp46, and DNAM1 receptors was reported on NK cells in peripheral blood and tumor tissue of metastatic CRC patients (Zhang et al., 2012; Rocca et al., 2013, 2016; Ferretti et al., 2020).

**TABLE 1:** A list of the most prominent bioactive molecules in tumor microenvironment and their effect on phenotype of NK cells and tumor cells undergoing epithelial to mesenchymal transition (* Regular fonts indicate inhibition of NK antitumor activity whereas italics indicate activation).

**Protein**	**Cell**	Effect*	**References**
TGF-β	Tumor	*MHC I downregulation*	Chen et al., 2015; Lorenzo-Herrero et al., 2018; Fedele and Melisi, 2020
		*E-cadherin downregulation*	Thuault et al., 2006, 2008; López-Soto et al., 2013; Chockley et al., 2018
		*MICA/B, ULBP1 upregulation*	López-Soto et al., 2006, 2013; Bedel et al., 2011; Huergo-Zapico et al., 2014
	NK	NKG2D, NCR, DNAM1 downregulation	Zhang et al., 2012; Rocca et al., 2013, 2016; Schiavoni et al., 2013; Konjević et al., 2019; Ferretti et al., 2020
IL-6	Tumor	PDL-1 upregulation	Wang H. B. et al., 2017
IL-8	Tumor	*E cadherin downregulation*	Li et al., 2012; Palena et al., 2012
IL-10	Tumor	*MHC I downregulation*	Stanilov et al., 2010; Konjević et al., 2019
	NK	NKG2D, NCRs downregulation	Schiavoni et al., 2013; Konjević et al., 2016
TNF	Tumor	*MICA downregulation*	Bedel et al., 2011; De Simone et al., 2015
NO	NK	CD16 downregulation	Stiff et al., 2018
L-kynurenine	NK	NKG2D, NCR downregulation	Della Chiesa et al., 2006; Konjević et al., 2016
Prostaglandine E2	NK	NKG2D, NCR downregulation	Harizi, 2013; Park et al., 2018
VEGF	Tumor	*E cadherin downregulation*	Yang et al., 2006; Bendardaf et al., 2019
	NK	NKG2D downregulation	Bruno et al., 2018
EGF	Tumor	*MHC I, E-cadherin downregulation*	Chen et al., 2015
lactate	NK	NKp46 downregulation	Husain et al., 2013
Matrix metalloproteinases	NK	CD16 shedding	Romee et al., 2013; Coppola et al., 2015
	Tumor	MICA/B, B7-H6, BAG6 shedding	Zhang et al., 2012; Schlecker et al., 2014; Iguchi-Manaka et al., 2016; Rusakiewicz et al., 2017; Zhao Z. et al., 2017; Molfetta et al., 2019

NK cell function is largely influenced by metabolic changes and nutrient availability in TME. Increased metabolic needs of proliferating tumor cells limit nutrient and oxygen availability and expose tumor-infiltrating NK cells to metabolites that drive their functional exhaustion. Tumor cells adapt to such environmental conditions by increasing consumption of glucose, upregulation of glycolysis and lactate production. The upregulation of hypoxia-inducible transcription factors (HIF)s, specifically HIF1α, induces the expression of glucose transporter (GLUT)1 and glycolytic enzymes, including lactate dehydrogenase (LDH)A (Hasmim et al., 2015). Increased accumulation of lactate in TME and low pH decrease NK cell cytotoxic activity and their ability to produce IFN-γ (Brand et al., 2016; Harmon et al., 2019; Domagala et al., 2020). Moreover, the accumulation of lactate in TME was reported to decrease the expression of activating receptor NKp46 (Husain et al., 2013) and impair energy metabolism by decreasing intracellular adenosine triphosphate (ATP) levels (Brand et al., 2016; Terrén et al., 2019).

Excessive glucose consumption by tumor cells deprives NK cells of their main fuel for metabolic processes. While resting NK cells metabolize glucose via oxidative phosphorylation (OXPHOS), activated NK cells upregulate glycolysis and glucose uptake and show increased expression of glucose transporter GLUT1 (Salzberger et al., 2018). In this sense, glycolytic pathway is facilitated during NK cell proliferation, cytotoxic activity, and IFN-γ production (Donnelly et al., 2014; Keppel et al., 2015). Signaling pathways that are crucial for metabolic reprogramming during NK cell activation are protein kinase mammalian target of rapamycin (mTOR), and transcription factors sterol regulatory element binding protein (SREBP) and cMyc (Donnelly et al., 2014; Assmann et al., 2017; Domagala et al., 2020). SREBP controls elevated metabolism of glucose to cytosolic citrate during NK cell functional response. Moreover, in advanced malignancies including CRC, increased level of SREBP1 inhibitor in TME has been correlated with NK cell dysfunction (Rossin et al., 2019). cMYC promotes OXPHOS and glycolysis by the upregulation of the glycolytic enzymes and expression of glucose transporters (Li et al., 2005). The glucose restriction in TME impairs NK cell function by upregulation of fructose-1,6-bisphosphatase (FBP1), an enzyme that inhibits glycolysis, in tumor infiltrating NK cells (Cong et al., 2018). Moreover, TGF-β which is considerably present in TME has been shown to inhibit both metabolism of glucose in NK cells and NK cell function. The proposed mechanism of action was through the inhibition of mTORC1 and not through the canonical TGF-β signaling pathway (Zaiatz-Bittencourt et al., 2018).

Adenosine is another suppressive mediator that is often present in TME. The hypoxic environment of solid tumors promotes the release of ATP and AMP which are by catalytic activity of ectonucleotidases CD39 and CD73 converted to AMP and adenosine, respectively (Ohta, 2016). Adenosine, via binding to adenosine A2A receptor (A2AR), inhibits metabolic activity (glycolytic capacity and OXPHOS) and effector functions of NK cells (Lokshin et al., 2006; Chambers et al., 2018).

NK cells compete for amino acids (glutamine, tryptophan, and arginine) with tumor cells and suppressive immune cells (MDSCs, TAMs) and CAFs in TME. Unlike tumors that consume amino acids to provide energy, NK cells utilize amino acids mainly for the maintenance of mTOR and cMyc cellular signaling that are necessary for NK cell functionality. Moreover, mTOR has been found to sustain the initial expression of cMyc while requiring glutamine for this process (Loftus et al., 2018). However, low concentration of arginine in TME impairs NK cell proliferation and IFN-γ production (Lamas et al., 2012). Suppressive immune cells by producing enzymes arginase, iNOS, and IDO deplete TME of arginine and tryptophan and increase the level of their metabolites NO and L-kynurenine. Furthermore, secreted NO decreases CD16 expression on NK cells and subsequently impairs antibody-dependent cellular cytotoxicity (ADCC) (Stiff et al., 2018).

Tumor cells undergoing EMT release IL-8 that stimulates tumor progression by supporting invasive phenotype of tumor cells (Li et al., 2012; Zhao Z. et al., 2017), angiogenesis and migration of immune cells to the tumor site and thus creating an inflammatory environment. IL-8, is a strong chemotactic factor for neutrophils which have protumorigenic and prometastatic functions as they induce intracellular adhesion molecule 1 (ICAM-1)-mediated binding of tumor cells to the surface of neutrophils and secretion of matrix metalloproteinases (MMPs) which remodel the extracellular matrix and favor tumor migration (Palena et al., 2012).

Tumor invasiveness is associated with increased synthesis and activity of MMPs. In this sense, it has been shown in CRC that EMT- related transcription factors Twist1/2 via binding to gene promoter induces transcription of MMP2 gene (Lu et al., 2018). Aside from enabling tumor invasiveness, MMP activity induces proteolytic cleavage of NK cell ligands from tumor cells leading to impaired NK cell recognition of tumor cells. Furthermore, MMP activity subsequently increases the level of soluble ligands that bind to activating NK cell receptors in the absence of target tumor cells and lead to NK cell dysfunction. The role of ligand shedding from tumor cells in the evasion of NK cell antitumor response and its clinical relevance have been shown for NKG2D (MICA/B, UL16-binding proteinsULBP-1,2), NKp30 (B7-H6, BAG6), DNAM1 (PVR-CD155) (Zhang et al., 2012; Schlecker et al., 2014; Iguchi-Manaka et al., 2016; Rusakiewicz et al., 2017; Zhao Y. et al., 2017; Molfetta et al., 2019; Table 1). Moreover, the persistent stimulation of activating receptors by their ligands induces post-activational receptor internalization and diminished NK cell activity as it was shown in experimental settings after co-culture with tumor cells, including CRC cells lines (Sconocchia et al., 2009; Rocca et al., 2013; Konjević et al., 2017b).

The expression CD16, a prominent NK cell cytotoxic receptor, was found to be decreased on NK cells in tumors, not only due to post-activational receptor internalization but also due to target cell induced activation of MMP, namely ADAM 17 (A disintegrin and metalloprotease 17), as shown during *in vitro* NK cell cultivation with tumor cells (Jewett and Tseng, 2011; Romee et al., 2013). Immunohistochemical data and data derived from co-culture experiments with CRC cell lines, also showed CD16 downregulation due to MMP proteolytic cleavage of this receptor from NK cell surface (Romee et al., 2013; Coppola et al., 2015). Moreover, as NK cells exert ADCC upon recognition of the Fc fragment of IgG bound to tumor cell surface by CD16, NK cells are involved in responses to monoclonal antibody (mAb) therapies such as cetuximab (anti-EGFR mAb), bevacizumab (anti-VEGF) mAb therapy are applied in treatment of metastatic CRC (Lorenzo-Herrero et al., 2018).

Chronic tumor cell ligand-NK cell receptor engagement leads to an exhausted NK cell phenotype characterized by upregulated PD-1 checkpoint immunoreceptor expression (Pesce et al., 2017). Recently, expression of several inhibitory checkpoint molecules that were initially considered to be inherent for T cells, TIM3, and TIGIT was shown on functionally exhausted NK cells in malignancies, including CRC (Beldi-Ferchiou and Caillat-Zucman, 2017).

## Conclusion

Immune cells represent an important factor that contributes to the EMT of tumor cells by supplying TME with bioactive molecules, enzymes, cytokines, chemokines, and physical interactions with tumor cells via their receptors. It has been well established that chronical inflammation in TME favors tumor growth and invasiveness and that cytokines, e.g., TGF-β, the most potent EMT inducer, induce synthesis of EMT promoting transcription factors. Chemokines and cytokines of the tumor-induced chronic inflammation, promote infiltration and differentiation of suppressive immune cells that further potentiate immunosuppression in TME. NK cells are one component of the innate immune system in the pool of diverse immune cells. The role of NK cells in the control of metastasis in CRC has been demonstrated in preclinical studies and tumor- infiltrating NK cells have been designated as a favorable prognostic factor in metastatic CRC patients.

Although, the cross-talk between immune cells in general and tumor cells favors the induction of EMT and inhibition of antitumor immune responses by tumor cells, there are some aspects of changes in immunogenicity of tumor cells during EMT of CRC that increase their susceptibility to NK cell cytotoxic lysis. Aside from the loss of MHC I expression on tumor cells during EMT, that makes them resistant to CD8^+^T and more susceptible to NK cells lysis, there are EMT-associated changes in the expression of ligands for NK cell receptors that make tumor cells more susceptible NK cell targets. In this sense, upregulation of tumor cell ligands MICA/B for activating NKG2D receptor, upregulation of adhesion CADM1 molecule, and downmodulation of E-cadherin ligand for inhibitory KLRG4 receptor, favor the elimination of tumor cell undergoing EMT by NK cells. However, EMT of tumor cells comprises a whole set of phenotypic states during their transition from epithelial to a fully mesenchymal phenotype which makes these interactions very complex. Moreover, the influence of immunosuppressive factors in TME by affecting expression of activating NK cell receptors and their multiple ligands on tumor cells, and upregulation of inhibitory immune checkpoint molecules on functionally exhausted NK cells have negative effects on the overall NK cell function. Furthermore, tumor infiltrating NK cells expressing PD-1, TIGIT, TIM3 are relevant for therapeutic targeting in the context of checkpoint blockade of PD-1/PD-L1 axis. In conclusion, recent studies have provided crucial information on the complex interactions of NK cells with CRC cells undergoing EMT and reveal new approaches for diagnostic and therapeutic applications that may improve patient management in the future.

## Author Contributions

AV, KM, NT, JZ, SC-B, and MČ wrote the manuscript. AV and KM designed and drew Figure 1, while AV, KM, and JZ designed and composed Table 1. All authors listed approved the manuscript for publication.

## Conflict of Interest

The authors declare that the research was conducted in the absence of any commercial or financial relationships that could be construed as a potential conflict of interest.

## Publisher’s Note

All claims expressed in this article are solely those of the authors and do not necessarily represent those of their affiliated organizations, or those of the publisher, the editors and the reviewers. Any product that may be evaluated in this article, or claim that may be made by its manufacturer, is not guaranteed or endorsed by the publisher.
